# Risk Analysis from the Burden of Lyme Disease (BOLD) Study

**DOI:** 10.3390/pathogens15090961

**Published:** 2026-09-09

**Authors:** Kate Halsby, Alexandra Loew-Baselli, Anna Moniuszko-Malinowska, Franc Strle, Johan Sanmartin Berglund, Ye Tan, Brad D. Gessner, Frederick J. Angulo, Juanita Edwards, Andreas Pilz, Elizabeth Begier, James H. Stark, on behalf of the BOLD Study Group

**Affiliations:** 1 Pfizer Ltd., Tadworth, Surrey KT20 7NS, UK; 2 Pfizer Corporation Austria GmbH, 1210 Vienna, Austria; alexandra.loew-baselli@pfizer.com (A.L.-B.); andreas.pilz@pfizer.com (A.P.); 3 Department of Infectious Diseases and Neuroinfections, Medical University of Białystok, 15-540 Bialystok, Poland; annamoniuszko@op.pl; 4 Department of Infectious Diseases, University Medical Centre Ljubljana, 1525 Ljubljana, Slovenia; franc.strle@kclj.si; 5 Department of Health, Blekinge Institute of Technology, 371 79 Karlskrona, Sweden; johan.sanmartin.berglund@bth.se; 6 Pfizer Inc., Cambridge, MA 02139, USA; ye.tan@pfizer.com (Y.T.); james.h.stark@pfizer.com (J.H.S.); 7 Pfizer Inc., Collegeville, PA 19426, USA; brad.gessner@epivac.net (B.D.G.); frederick.j.angulo@pfizer.com (F.J.A.); juanita.edwards@pfizer.com (J.E.); 8 Pfizer Biopharma Group, D04 K7N3 Dublin, Ireland; elizabeth.begier@pfizer.com

**Keywords:** Lyme borreliosis, Lyme disease, risk factors, Europe, burden of disease

## Abstract

Lyme borreliosis (LB) is endemic in many European countries. The prospective, epidemiological Burden of Lyme Disease (BOLD) study used unified case definitions to characterise LB burden in Europe. A retrospective case–control study was nested within the BOLD study to better understand LB risk factors. LB patients were evaluated at 14 healthcare practices in high-LB-incidence areas in six European countries: the Czech Republic, Germany, Poland, Slovakia, Slovenia, and Sweden between April 2021 and December 2022. Enrolled cases and age-group-matched controls were interviewed about potential risk factors, and multivariate regression analysis was used to analyse the responses. Patients with LB were more likely than matched controls to live in neighbourhoods with >50% woods, to have a garden that is composed of less than half dense trees, and to have a garden visited by wild animals. Characteristics associated with lower LB risk included being male and not having a bird feeder. The results are suggestive of risk associated with edge-of-the-forest areas, aligning with the previous literature on the increased risk of LB with forest fragmentation. Our findings support the importance of landscape structure and human–environment interactions in shaping LB risk.

## 1. Introduction

Lyme borreliosis (LB) is the most common vector-borne disease in Europe and represents a growing public health concern [1]. LB is a bacterial infection caused by *Borrelia burgdorferi sensu lato* complex species, primarily transmitted through the bite of infected *Ixodes* ticks. A quarter of the European population is estimated to reside in an LB-endemic area [2,3]. Over the past few decades, as ecological and environmental conditions have changed, the geographic range and incidence of LB have increased [4,5]. Warmer environmental temperatures and increased humidity have resulted in a greater abundance of ticks, extended the length of active tick seasons, and allowed tick population expansion into previously nonendemic regions [6,7].

LB risk is determined by two main areas: environmental risk and human behaviour. Forest cover, habitat fragmentation, and biodiversity influence tick density and host availability, with forested areas and urban green spaces supporting higher densities of infected ticks [6,8,9,10,11]. Changes in land use, including reforestation, agricultural practices, and urban expansion have been linked to altered tick distribution and increased human–tick interactions [3,11,12]. Human behaviours are also central determinants of exposure risk. Individuals engaged in outdoor occupations such as forestry or agriculture or those who regularly partake in recreational activities such as hiking, camping, and foraging are at increased risk of tick bites and subsequent infection [3,12].

Until recently there was a lack of multi-country epidemiological studies in Europe, hindering accurate understanding of LB burden at an above-country level. Recently, results from the Burden of Lyme Disease (BOLD) study [13] have been published and provide a comprehensive evaluation of the current burden of medically attended LB across Europe [14,15,16]. The BOLD study was a prospective epidemiological study using standardised case definitions and active surveillance to better characterise LB burden at high-endemic sites across Europe to assist in the identification of potential trial sites for assessment of a candidate outer surface protein A (OspA) Lyme disease vaccine. Results from BOLD have been previously published, including the incidence of LB (under review) [17], symptoms and manifestations [14], treatments [14], patient-reported outcome measures [15] and post-treatment Lyme disease syndrome (PTLDS) in the study [16]. This manuscript describes a nested case–control study which was an exploratory analysis within the context of the BOLD study. The analysis aimed to identify risk factors potentially associated with LB (e.g., time outdoors, pets, occupational and leisure exposures) in a population of individuals across six European countries (the Czech Republic, Germany, Poland, Slovakia, Slovenia and Sweden). A comprehensive understanding of these risk factors is essential for informing targeted prevention strategies, improving surveillance, and reducing LB burden across Europe.

## 2. Materials and Methods

### 2.1. Study Design and Setting

The detailed BOLD study methodology has been published elsewhere [13]. In brief, the analysis herein builds upon a prospective, active surveillance, and epidemiological study conducted at 14 general practices/primary care practices located in areas of high-LB incidence in 6 European countries: the Czech Republic (3 sites), Germany (5 sites), Poland (2 sites), Slovakia (2 sites), Slovenia (1 site), and Sweden (1 site). Primary care clinics with adequate clinical research infrastructure and a historical incidence of LB of at least 500 per 100,000 population per year were selected for inclusion. Active surveillance for suspected LB cases with optional study enrolment began after each site’s activation date (April–July 2021) and lasted 12 months (through April–July 2022). Thereafter, active surveillance without the opportunity for enrolment continued until the end of 2022. Age-matched controls were identified for each enrolled LB case, selected at random from the population associated with the primary care practice of the LB case (referred to here as the “practice panel”). Cases attended up to 5 study visits over two years, and controls had up to two contact points (day 1 and month 16–18). This analysis focuses on the first study visit for both cases and controls.

The local ethics committee for each participating site (Appendix B) approved this study, and investigation was conducted in accordance with legal and regulatory requirements, the International Ethical Guidelines for Biomedical Research Involving Human Participants, the International Council for Harmonisation Good Clinical Practice Guideline, and the Declaration of Helsinki. Written informed consent was obtained for each participant before starting study-specific activities.

### 2.2. LB Cases and Control Participants

The BOLD study identified medically attended LB cases (individuals who sought medical care at their participating clinic and received a clinical diagnosis for erythema migrans or a clinical and laboratory diagnosis for disseminated disease, Table S1). Patients had to meet all of the following inclusion criteria to be enrolled in the consented portion of the study (there were no exclusion criteria):

1. Member of participating patient practice.
2. Suspected or confirmed newly diagnosed LB during the enrolment period, regardless of timing of infection.
3. Evidence of a personally signed and dated informed consent/assent (when age-appropriate and per local requirements) document.

Between 6 and 10 control participants in the age group of the corresponding enrolled LB case were selected at random from the practice panel of the primary care practice of the LB case and then contacted if they were willing to sign the informed consent document and enrol in the study. The aim was to enrol at least four controls per LB case. This was done as soon as possible after LB case enrolment and diagnosis. The age-matching was carried out by year for participants between 0 and 19 years of age and by 5-year age groups thereafter (e.g., 20–24 years, 30–34 years). Control participants had to meet both of the following inclusion criteria to be eligible for inclusion in the study:

1. Member of participating patient practice at the time of associated LB case diagnosis.
2. Evidence of a personally signed and dated informed consent/assent (when age-appropriate and per local requirements) document.

Furthermore, control participants were excluded if they had active LB in the last 90 days. If a control later became a suspected LB case, they were retained as a control if they (1) did not display any LB-related symptoms at the time of enrolment as a control and (2) showed no other evidence of infection, such as seroconversion within 30 days of enrolment as a control. Seven subjects who developed LB after consenting as controls were excluded from the control analysis under these rules. Only a single subject who presented with a newly diagnosed erythema migrans after completing their control visit and who had a negative Lyme serology at the time of enrolment as a case (confirming no preexisting LB infection) was retained as both a control and, subsequently, a case.

### 2.3. Nested Case–Control Risk Factor Analysis

Information on potential risk factors for LB was collected by interview from all enrolled LB cases and matched controls. The 10 to 15 minute survey (Appendix C) was administered in clinic at visit 1 (date of initial study visit) for cases, and either over the phone, by post, or in clinic at the time of contact point 1 (date of initial control enrolment) for controls. It included questions on education, occupation, outdoor activities, residential characteristics (e.g., tree coverage), and lifestyle and outdoor activities. Questions were developed to capture data on covariates that were potentially clinically relevant based on the natural history of the disease and the scientific literature. Adult respondents completed the survey for themselves. Caregivers completed the survey for children. The questionnaire was translated for each country and reviewed by the relevant ethics committees. Detailed instructions were provided to site personnel for supervision of the questionnaire responses. Missing data were not imputed. Univariate and multivariate conditional logistic regression was performed to calculate odds ratios (ORs) and adjusted odds ratios (aORs) based on the presence (cases) or absence (controls) of LB. Risk factors for the multivariate analyses were based on stepwise regression analyses, with entry into the model at *p* < 0.1 and retention at *p* < 0.2. Sex was retained regardless of univariate results. Analysis was conducted using Statistical Analysis System (SAS) software (version 9.4).

## 3. Results

There were 315 enrolled medically attended LB cases and 1379 enrolled controls; their demographics and clinical characteristics have previously been reported (Table S2) [14]. Of these, two cases and four controls did not complete the risk factor questionnaire; one case had a missing response for the variable ’Neighbourhood greater than 50% woods‘, and six controls had a missing response for the variable ’Occupation’. The distribution of potential risk factors among the controls, stratified by country, is listed in Table S3.

Risk factors associated with an increased risk of LB were identified from the multivariate analyses (Table 1) included residing in neighbourhoods with >50% woods (OR 2.42, 95% confidence interval [CI] 1.82–3.20; aOR 2.20, 95% CI 1.61–2.99), having a garden with less than half dense trees (OR 2.01, 95% CI 1.48–2.72; aOR 1.83, 95% CI 1.27–2.64), garden being visited by wild animals (OR 1.88, 95% CI 1.37–2.57; aOR 1.46, 95% CI 1.02–2.09), and the response ‘no garden’ to the question ‘Garden visited by wild animals’ (aOR 4.39, 95% CI 1.87–10.29). Characteristics associated with lower LB risk included being male (OR 0.74, 95% CI 0.57–0.96; aOR 0.70, 95% CI 0.53–0.93) and not having a bird feeder (OR 0.35, 95% CI 0.20–0.60; aOR 0.24, 95% CI 0.11–0.50). Summer activity (aOR 0.98, 95% CI 0.97–0.99) was marginally protective whilst autumn activity showed a slightly increased risk of LB (OR 1.38, 95% CI 1.04–1.81; aOR 1.03, 95% CI 1.01–1.04).

The following categories showed an increased risk of LB in the univariate analysis only: having a garden bordering on woods (OR 1.81, 95% CI 1.33–2.47), having a garden with half or more dense trees (OR 1.85, 95% CI 1.26–2.72), leisure activities of foraging (OR 1.81, 95% CI 1.34–2.44), gardening (OR 1.37, 95% CI 1.02–1.86) and hunting (OR 1.91, 95% CI 1.13–3.24). Additionally, winter activity was associated with lower LB risk in the univariate analysis alone (OR 0.74, 95% CI 0.57–0.97).

## 4. Discussion

This analysis of a nested case–control study used data from the multi-country BOLD study to better understand risk factors associated with LB in Europe. The case–control analysis highlights factors that are suggestive of risk associated with edge-of-the-forest areas where human activity is more likely to occur than in densely forested areas: residing in neighbourhoods with >50% woods and having a garden that is composed of less than half dense trees. These findings align with the previous literature on forest fragmentation, which shows the highest LB risk occurring at 20% forest cover [18,19]. Fragmented forests and green areas can influence landscape connectivity, with these spaces being more likely to be visited by animals that can carry ticks, such as roe deer [6,20]. Our study also found that having a garden visited by wild animals was an LB risk factor. Interestingly, for this question, having ‘no garden’ was also a risk factor. It is possible that people without personal gardens spend more time in forested areas.

The analysis gave complex results for seasonal risk, with a marginally protective effect seen in summer (Jul–Sept), and a slightly increased risk of LB in autumn (Oct–Dec). The typical presentation of LB cases shows a peak in early summer [21]. There may be some time delay between exposure and disease presentation, especially for disseminated cases. Leisure activities showed an association with LB risk in the univariate analysis, but not in the multivariate model. The reason for this is unclear, but there may have been confounding with the seasonal time spent outdoors.

Strengths of the BOLD study included using consistent LD case definitions from high-incidence areas across six European countries, and selecting controls contemporaneously from within the same GP practice allowed for control of the endemicity of the surrounding geographic region and thus the assessment of the impact of characteristics of the participant’s home, garden and behaviour on risk.

The analysis was restricted by some limitations. A couple of categories had small sample sizes, making interpretation difficult, e.g., no bird feeder (*n* = 18 cases; aOR 0.24; 95% CI, 0.11–0.50), and response ‘no garden’ to the question ‘is your garden visited by wild animals’ (*n* = 20 cases; aOR 4.39; 95% CI, 1.87–10.29). An additional limitation is that the study pools data from six European countries where societal exposure to risk factors for LB may differ (see Table S3). Country-specific multivariate analyses were not possible due to small numbers of cases in 4/6 countries. The six countries will have experienced different weather and tick seasons over the study period, and aggregating the data may therefore have diluted any seasonal information. Also, countries contributing more cases will have had a greater impact on study results than countries contributing fewer cases. Individual country models were considered as a way of overcoming these limitations during the analysis phase, but case numbers were too small.

As ecological and environmental changes expand the geographical range of endemic LB areas across Europe, the present findings provide further evidence that spatial configuration of landscape at a local level plays a critical role in shaping exposure risk. The analysis identified environmental characteristics associated with forest-edge environments as key risk factors, complementing the existing literature and highlighting that these transitional zones between forest and human-inhabited spaces represent heightened exposure settings. Enhanced understanding of these risk factors may help inform surveillance and prevention efforts, ultimately working to reduce LB burden across Europe.

## 5. Conclusions

Risk factor findings from the BOLD study, including data across six European countries, are consistent with prior reports. Specifically, individuals residing in neighbourhoods with more than 50% woodland cover and having partially wooded gardens were both associated with elevated LB risk, highlighting these interface areas are key exposure zones. Overall, these findings underscore the importance of landscape structure and human–environment interactions in shaping LB risk, informing surveillance and prevention strategies.

## Figures and Tables

**Table 1:** Univariate and Multivariate Associations Between Study Variables and LB Risk.

Studied Variable/Category	Cases, n (%) (N = 315)	Controls, n (%) (N = 1379)	Univariate Odds Ratio (95% CI)	Adjusted Odds Ratio (95% CI)
Demographics				
Biological sex				
Female	179 (56.8)	700 (50.8)	Reference	Reference
Male	136 (43.2)	679 (49.2)	0.74 (0.57–0.96) *	0.70 (0.53–0.93) *
Race				
White	315 (100.0)	1376 (99.8)	Reference	
Non-White	0 (0.0)	3 (0.2)	0.0 (0.00–1.00)	
Highest education level				
Secondary school or less ^b^	206 (65.4)	962 (69.8)	Reference	
University/graduate school	107 (34.0)	413 (29.9)	1.16 (0.88–1.53)	
Missing	2 (0.6)	4 (0.3)	2.17 (0.24–19.82)	
Occupation				
Indoor occupation	127 (40.3)	595 (43.1)	0.93 (0.62–1.40)	
Outdoor occupation ^a^	50 (15.9)	161 (11.7)	1.39 (0.87–2.22)	
Student	7 (2.2)	36 (2.6)	0.56 (0.16–1.96)	
No occupation ^b^	126 (40.0)	559 (40.5)	Reference	
Missing or unknown	5 (1.6)	28 (2.0)	0.80 (0.28–2.29)	
Residence				
Location				
Urban	78 (24.8)	379 (27.5)	Reference	
Rural	150 (47.6)	627 (45.5)	1.42 (0.89–2.28)	
Small town	79 (25.1)	349 (25.3)	1.18 (0.73–1.92)	
Missing or unknown	8 (2.5)	24 (1.7)	2.22 (0.87–5.70)	
Fencing around garden				
Yes	217 (68.9)	909 (65.9)	1.06 (0.78–1.45)	
No	75 (23.8)	338 (24.5)	Reference	
No garden ^b^	20 (6.3)	121 (8.8)	0.75 (0.42–1.35)	
Missing or unknown	3 (1.0)	11 (0.8)	1.05 (0.25–4.42)	
Garden borders on woods				
Yes	138 (43.8)	426 (30.9)	1.81 (1.33–2.47) *	
No	150 (47.6)	800 (58.0)	Reference	
No garden ^b^	23 (7.3)	145 (10.5)	0.83 (0.50–1.37)	
Missing or unknown	4 (1.3)	8 (0.6)	2.49 (0.64–9.74)	
Garden visited by wild animals				
Yes	150 (47.6)	476 (34.5)	1.88 (1.37–2.57) *	1.46 (1.02–2.09) *
No	129 (41.0)	718 (52.1)	Reference	Reference
No garden ^b^	20 (6.3)	121 (8.8)	0.90 (0.52–1.56)	4.39 (1.87–10.29) *
Missing or unknown	16 (5.1)	64 (4.6)	1.30 (0.71–2.38)	1.15 (0.58–2.28)
How much of garden is dense trees				
No woods ^b^	83 (26.3)	526 (38.1)	Reference	Reference
Less than half of it	170 (54.0)	604 (43.8)	2.01 (1.48–2.72) *	1.83 (1.27–2.64) *
Half or more	58 (18.4)	213 (15.4)	1.85 (1.26–2.72) *	1.42 (0.91–2.23)
Missing or unknown	4 (1.3)	36 (2.6)	0.81 (0.27–2.50)	0.44 (0.09–2.03)
Neighbourhood greater than 50% woods				
Yes	145 (46.0)	373 (27.0)	2.42 (1.82–3.20) *	2.20 (1.61–2.99) *
No	152 (48.3)	892 (64.7)	Reference	Reference
Missing or unknown ^b^	18 (5.7)	114 (8.3)	0.84 (0.47–1.50)	0.90 (0.44–1.82)
Bird feeder within reach of wild animals				
Yes	117 (37.1)	427 (31.0)	1.13 (0.84–1.50)	0.88 (0.64–1.21)
No	176 (55.9)	762 (55.3)	Reference	Reference
No bird feeder ^b^	18 (5.7)	179 (13.0)	0.35 (0.20–0.60) *	0.24 (0.11–0.50) *
Missing or unknown	4 (1.3)	11 (0.8)	1.48 (0.40–5.52)	3.33 (0.62–17.81)
Lifestyle and Outside Activities				
Pets spend time outside				
Yes	154 (48.9)	625 (45.3)	1.15 (0.88–1.49)	
No ^b^	158 (50.2)	749 (54.3)	Reference	
Missing or unknown	3 (1.0)	5 (0.4)	2.96 (0.52–16.87)	
Outside leisure activities				
Biking	161 (51.1)	613 (44.5)	1.28 (0.98–1.67)	
Camping	44 (14.0)	180 (13.1)	0.92 (0.63–1.37)	
Fishing	22 (7.0)	111 (8.0)	0.75 (0.46–1.21)	
Foraging	159 (50.5)	526 (38.1)	1.81 (1.34–2.44) *	
Gardening	228 (72.4)	900 (65.3)	1.37 (1.02–1.86) *	
Hiking	208 (66.0)	837 (60.7)	1.25 (0.94–1.65)	
Hunting	25 (7.9)	56 (4.1)	1.91 (1.13–3.24) *	
Sports	108 (34.3)	387 (28.1)	1.32 (0.98–1.78)	
Picnicking	96 (30.5)	390 (28.3)	0.99 (0.73–1.34)	
Outdoor play (children)	81 (25.7)	412 (29.9)	0.78 (0.58–1.06)	
Other	25 (7.9)	138 (10.0)	0.71 (0.45–1.12)	
None ^b^	4 (1.3)	39 (2.8)	0.46 (0.16–1.33)	
Missing	2 (0.6)	4 (0.3)	N/A ^c^	
Time spent outside by season ^d,e^, mean (hours per week) (SD)				
Spring—leisure activity	19.2 (12.9)	18.7 (14.2)	1.16 (0.88–1.51)	
Spring—occupational activity	5.9 (13.3)	4.7 (11.1)		
Summer—leisure activity	27.3 (17.1)	27.6 (18.5)	1.04 (0.79–1.36)	0.98 (0.97–0.99) *
Summer—occupational activity	7.2 (16.2)	6.0 (13.6)		
Autumn—leisure activity	20.2 (13.4)	19.1 (14.6)	1.38 (1.04–1.81) *	1.03 (1.01–1.04) *
Autumn—occupational activity	6.3 (14.3)	4.6 (10.8)		
Winter—leisure activity	12.2 (9.5)	11.1 (10.0)	0.74 (0.57–0.97) *	
Winter—occupational activity	4.6 (11.7)	3.4 (8.5)		

CI = Confidence interval; LB = Lyme borreliosis; NE = not evaluated; SD = standard deviation. Note: Limited to cases with associated controls enrolled. The odds ratios were point estimates with the corresponding 95% confidence limits from a logistic regression model. This table presents the factors from the final model based on stepwise regression analysis. Entry to model was *p* < 0.1; retention was *p* < 0.2; blank cells indicate variables that did not meet this threshold. Grey shading indicates broad question categories. * Confidence intervals do not cross 1 (before rounding). ^a^ Outdoor occupations included construction, farming, forestry, land surveying, landscaping, park or wildlife management, railroad work, utility line work, and other outdoor work. ^b^ Includes “Not applicable.” ^c^ No comparison group to calculate the OR. Data was missing for the whole category of outside leisure activities, not for each activity. ^d^ Seasons were defined as: spring (April to June), summer (July to September), autumn (October to December), winter (January to March). ^e^ Seasonal variables were combined in the regression analysis (occupational + summer leisure).

## Data Availability

Upon request, and subject to review, Pfizer will provide the data that support the findings of this study. Subject to certain criteria, conditions and exceptions, Pfizer may also provide access to the related individual de-identified participant data. See https://www.pfizer.com/science/clinical-trials/data-and-results (accessed on 6 September 2026) for more information.

## Supplementary Materials

The following supporting information can be downloaded at https://www.mdpi.com/article/10.3390/pathogens15090961/s1, Table S1: BOLD case definitions; Table S2: Demographics and clinical characteristics of enrolled medically attended LB cases and controls; Table S3: Distribution of risk factors for controls, by country.

## Abbreviations

The following abbreviations are used in this manuscript:

aOR	Adjusted odds ratio
BOLD	Burden of Lyme Disease
CI	Confidence interval
LB	Lyme borreliosis
NE	Not evaluated
SAS	Statistical Analysis System
SD	Standard deviation

## Appendix A

### Investigators of the Burden of Lyme Disease (BOLD) Study Group

- Dr. Lenka Dybova; Doktor Brno, S.R.O.; Brno, Czech Republic
- Dr. Miroslav Pavlasek; ADMED, S.R.O.; Ceske Budejovice, Czech Republic
- Dr. Daniela Verdanova; Ordinace praktickeho lekare pro deti a dorost, Jindrichuv Hradec, Czech Republic
- Dr. Joachim Weimer; Private Practice of Dr. Joachim Weimer; Reinfeld, Germany
- Dr. Christine Kosch; Private Practice of Dr. Christine Kosch; Pirna, Germany
- Dr. Kai Mehltretter; Velocity Clinical Research; Leipzig, Germany
- Dr. Hans-Detlev Stahl; AmBeNet GmbH; Leipzig, Germany
- Dr. Viliam Cibik, MUDr; Viliam Cibik, PhD, S.R.O.; Pruske, Slovakia
- Dr. Dagmar Zakova, MUDr; Zakova, s.r.o; Ambulancia vnutorneho lekarstva, Trencin, Slovakia
- Dr. Barbara Nieradko-Iwanicka; Lubelskie Centrum Diagnostyczne, Swidnik, Poland and Medical University of Lublin, Lublin, Poland
- Dr. Dorota Bielska; Akademicka Praktyka Medycyny Rodzinnej, Bialystok, Poland
- The BOLD study group also included Drs Berglund, Moniuszko-Malinowska, and Strle who are listed separately as co-authors.

## Appendix B

**Table A1:** Ethics Committee Approval Information.

Country	Ethics Committee	Initial Approval Date	Initial Approval Code
Czech Republic	Eticka komise IKEM a FTN Fakultni Thomayerova nemocnice Videnska 800 Praha 4—Krc, 140 59	16 February 2021	12/21 + 4217/21
Germany	Ethik-Kommission bei der Landesärztekammer Hessen Hanauer Landstrasse 152 Frankfurt am Main, 60314	12 March 2021	2021-2414-zvBO
Germany	Ethikkommission bei der Ärztekammer Schleswig-Holstein Bismarckallee 8–12 Bad Segeberg, 23795	12 March 2021	034/21 m
Germany	Ethikkommission bei der Sächsischen Landesärztekammer Schützenhöhe 16 Dresden, SACHSEN 01099	12 March 2021	EK-BR-22/21-1
Poland	Komisja Bioetyczna przy Lubelskiej Izbie Lekarskiej w Lublinie ul. Chmielna 4 Lublin, 20-079	1 February 2021	140/2021/KB/VIII
Poland	Komisja Bioetyczna przy Uniwersytecie Medycznym w Bialymstoku ul. Jana Kilinskiego 1 Bialystok, 15-089	25 March 2021	APK.002.169.2021
Poland	Komisja Bioetyczna przy Okregowej Izbie Lekarskiej z siedziba w Bialymstoku ul. Swietojanska 7 Bialystok, 15-082	21 April 2021	12/2021/VIII
Slovakia	Eticka Komisia Trencianskeho samospravneho Karja K dolnej stanici 7282/20A Trencin, 911 01	10 February 2021	No code provided—the approval references Pfizer’s protocol number: C4601002
Slovenia	Komisija Republike Slovenije za medicinsko etiko Stefanova 5 Ljubljana, 1000	31 May 2021	0120-53/2021-4
Sweden	Etikprövningsmyndigheten Box 2110 Uppsala, 750 02	5 May 2021	2021-00604

## Appendix C

### Annotated Lyme Disease Risk Factor Questionnaire

You are being asked to complete this Risk Factor Questionnaire as part of your participation in this study. The information you provide in this questionnaire will help Pfizer (the “sponsor”) learn more about the risk factors (e.g., what puts you at risk) for Lyme disease. The study team will work with you to answer any questions you may have about completing this questionnaire.

	**Question**	**Response**	
1	Date of Assessment	(DD/MM/YYYY)	
2	Method of Administration	1. Self 2. Assisted	
3	What is the highest level of education achieved?	1. University/Graduate school 2. Primary and Secondary school 3. Not Applicable	
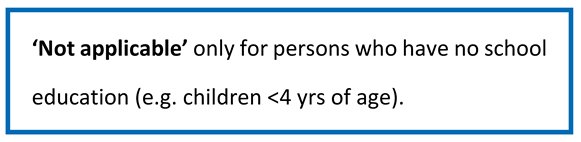
4	Do you have any house pets that spend time outside? For the purpose of this study, a house pet is defined as an animal (such as a cat or dog) that is allowed to spend some time inside your home between May and October.	1. Yes (specify): _________________________ 2. No 3. Unknown 4. Not Applicable	
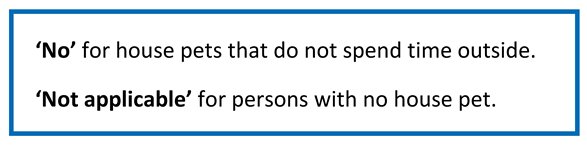
5	What is your current occupation?	1. Construction 2. Landscaping 3. Forestry 4. Brush clearing 5. Land surveying 6. Farming 7. Railroad work 8. Oil field work 9. Utility line work 10. Park or wildlife management 11. Other outdoor work 12. Other non-outdoor work 13. Student 14. Not applicable 15. Unknown	
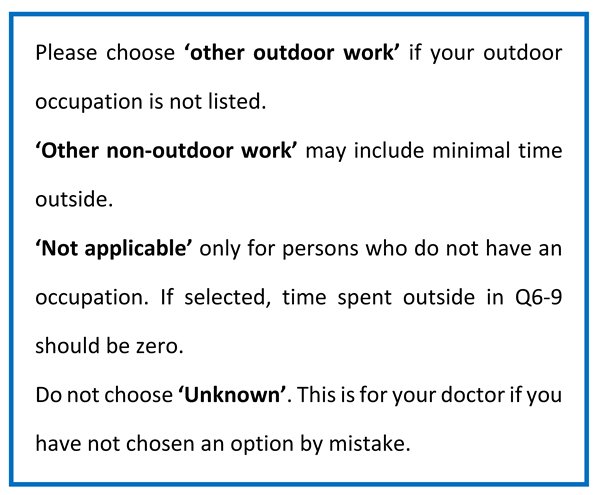
6	How many hours on average per week do you spend outside for your occupation by season? Spring:	Time Spent Outside _______	Unit Hours per week
7	Summer:	_______	Hours per week
8	Autumn:	_______	Hours per week
9	Winter:	_______	Hours per week
10	What leisure time activities do you or your child engage in outside (select all that apply)?	1. Gardening 2. Foraging 3. Hiking 4. Hunting 5. Fishing 6. Biking 7. Picnicking 8. Sports 9. Outdoor play (children) 10. Camping 11. Not Applicable 12. Other (Please specify below)	
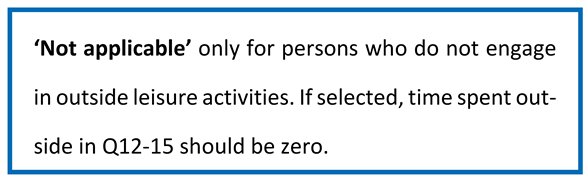
11	If you selected ‘Other’ in Question 10 above, please specify.	____________________	
12	How many hours on average per week do you or your child spend outside for leisure time by season? Spring:	Time Spent Outside _______	Unit Hours per week
13	Summer:	_______	Hours per week
14	Autumn:	_______	Hours per week
15	Winter:	_______	Hours per week
16	Does your garden (house or grounds of apartment), have fencing around it or parts of it?	1. Yes 2. No 3. Unknown 4. Not Applicable	
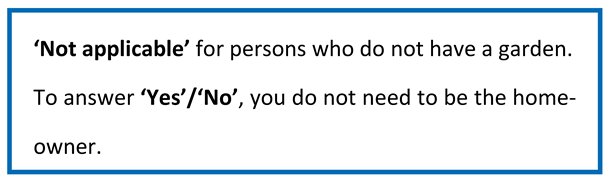
17	Do you have a bird feeder that is within reach of wild animals (e.g., deer, foxes, badgers, rodents)?	1. Yes 2. No 3. Unknown 4. Not Applicable	
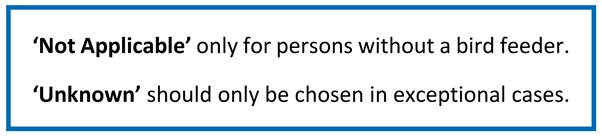
18	Is your garden (house or grounds of apartment) regularly visited by wild animals (e.g., deer, foxes, badgers, rodents)?	1. Yes 2. No 3. Unknown 4. Not Applicable	
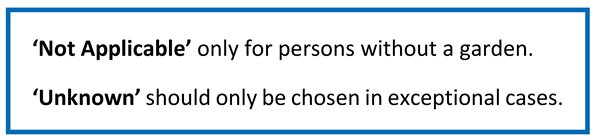
19	How much of your garden is covered in dense trees?	1. All of it 2. Greater than half of it 3. About half of it 4. Less than half of it 5. No woods on property 6. Unknown 7. Not Applicable	
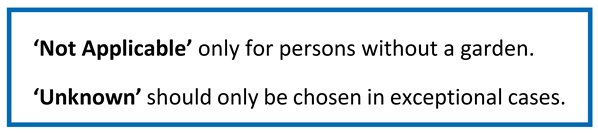
20	Does your garden border on woods including property not owned by you or your landlord?	1. Yes 2. No 3. Unknown 4. Not Applicable	
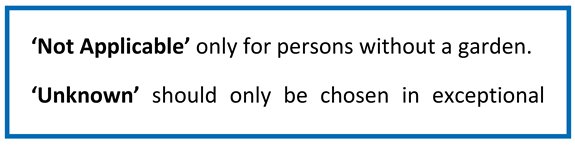
21	Does your neighbourhood consist of greater than 50% forest or wooded area?	1. Yes 2. No 3. Unknown 4. Not Applicable	
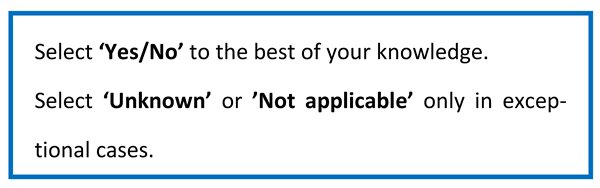
22	How would you describe the location of your home?	1. Large urban areas (settlements with 125 K+ people) 2. Other urban areas (settlements with 10–125 K people) 3. Accessible small towns (settlements of 3–10 K people, <30 min drive to town of 10 K + people) 4. Remote small towns (settlements of 3–10 K people, >30 min drive to town of 10 K + people) 5. Accessible rural (areas with <3 K people, and <30 min drive to town of 10 K + people) 6. Remote rural (areas with <3 K people and >30 min drive to town of 10 K + people) 7. Unknown	
