# Supplementary material for: Risk Analysis from the Burden of Lyme Disease (BOLD) Study
Source: Pathogens. 2026 Sep 9;15(9):961. doi: 10.3390/pathogens15090961 (PMC13610116; doi:10.3390/pathogens15090961)
Supplement: Supplementary file 1 [file pathogens-15-00961-s001.zip › pathogens-4466267-supplementary.pdf]

pathogens

MDPI

Supplementary Tables

Table S1: BOLD case definitions

Presentation[a]	Sign/Symptom	Laboratory Diagnostic(s)[b]
Erythema migrans	Characteristic red or bluish-red patch, with or without central clearing	Positive IgG/IgM on serum Bbsl antibody testing OR<br>Positive PCR of Bbsl result from biopsy OR<br>Positive culture of Bbsl from biopsy
Borrelial lymphocytoma	Painless bluish-red nodule or plaque, usually on ear lobe, ear helix, nipple, or scrotum	
Acrodermatitis chronica atrophicans	Long-standing red or bluish-red lesions, usually on the extensor surfaces of extremities. Initially doughy swelling. Possible skin induration and fibroid nodules over bony prominences	
Lyme neuroborreliosis	Meningo-radiculoneuritis (Bannwarth syndrome), facial palsy, meningitis, encephalomyelitis, OR cerebral vasculitis	Intrathecal Bbsl IgM and/or IgG antibodies OR<br>Positive intrathecal *Borrelia* antibody index (CSF versus serum) reflecting intrathecal Bbsl antibody production OR<br>Positive PCR of Bbsl result from CSF OR<br>Positive culture of Bbsl from CSF
Lyme carditis	Acute onset of high degree atrioventricular conduction disturbances, rhythm disturbances, myocarditis, OR pancarditis	Positive IgG/IgM on serum Bbsl antibody testing
Lyme arthritis	Marked swelling in 1 or few large joints, most often the knee	Positive IgG/IgM on serum Bbsl antibody testing OR<br>Positive PCR of Bbsl result from synovial fluid or tissue OR<br>Positive Culture of Bbsl from synovial fluid or tissue
LB ocular manifestations	Conjunctivitis, uveitis, papillitis, episcleritis, OR keratitis	Positive IgG/IgM on serum Bbsl antibody testing OR<br>Positive PCR of Bbsl result from ocular fluid OR<br>Positive culture of Bbsl from ocular fluid

Bbsl = Borrelia burgdorferi sensu lato; BL = borrelial lymphocytoma; BOLD = Burden of Lyme Disease; CSF = cerebrospinal fluid; EM = erythema migrans; IgG = immunoglobulin G; IgM = immunoglobulin M; LB = Lyme borreliosis; PCR = polymerase chain reaction.

https://doi.org/10.3390/pathogens15090961

[a] All presentations were considered disseminated Lyme manifestations and required laboratory confirmation, except for EM (singular lesion) and BL; EM or BL was only considered disseminated if there were multiple lesions and laboratory confirmation. Other EM cases could be diagnosed based on clinical symptoms alone. Disseminated cases that presented with more than one manifestation including an EM rash could be diagnosed clinically without laboratory confirmation.

[b] At least one positive standard of care diagnostic test result was required for laboratory confirmation of medically attended LB.

https://doi.org/10.3390/pathogens15090961

Table S2. Demographics and clinical characteristics of enrolled medically attended LB cases and controls

	**Cases (N = 315)**	**Controls (N = 1379)**
**Country, n**		
Czech Republic	32	141
Germany	42	165
Poland	9	34
Slovakia	107	566
Slovenia	21	110
Sweden	104	363
**Sex, n (%)**		
Female	179 (56·8)	700 (50.8)
Male	136 (43·2)	679 (49.2)
**Age, years**		
Mean (SD)	54·5 (16·6)	55.4 (16.5)
Median (Q1, Q3)	57 (45, 67)	57.0 (45.0, 68.0)
**Race, n (%)**[a]		
White	315 (100·0)	1376 (99.8)
Asian	0	3 (0.2)
**Manifestations, n (%)**		
Erythema migrans	210 (66·7)	N/A
Lyme neuroborreliosis	2 (0·6)	N/A
Lyme arthritis	80 (25·4)	N/A
Lyme carditis	1 (0·3)	N/A
Other[b]	13 (4·1)	N/A
> 1 Manifestation[c]	9 (2·9)	N/A

LB = Lyme borreliosis; N/A = not applicable; Q1 = quartile 1; Q3 = quartile 3; SD = standard deviation.

[a] No individuals had Black or African American race or were multiracial.

[b] Other = LB diagnosis without established LB clinical manifestation as defined in Table S-1, e.g., systemic symptoms only.

[c] Erythema migrans and Lyme arthritis (n = 6), erythema migrans and Lyme carditis (n = 1), erythema migrans and Lyme neuroborreliosis (n = 1), and Lyme arthritis and pronounced unspecific general symptoms (n = 1).

Table S3. Distribution of risk factors for controls, by country

Risk Factor	Czech Republic (N = 141)	Germany (N = 165)	Poland (N = 34)	Slovakia (N = 566)	Slovenia (N = 110)	Sweden (N = 363)	Total (N = 1379)
Biological sex							
Female	79	100	19	275	64	163	700
Male	62	65	15	291	46	200	679
Race							
White	140	164	34	566	110	362	1376
Non-White	1	1	0	0	0	1	3
Highest education level							
Secondary school or less [b]	109	108	13	430	70	232	962
University/graduate school	32	53	21	136	40	131	413
Missing	0	4	0	0	0	0	4
Occupation							
Indoor occupation	52	46	11	285	61	140	595
Outdoor occupation [a]	19	7	5	75	15	40	161
Student	16	11	0	3	1	5	36
No occupation [b]	51	97	18	196	22	175	559
Missing or unknown	3	4	0	7	11	3	28
Residence							
Location							
Urban	93	81	24	154	16	11	379
Rural	23	31	6	342	59	166	627
Small town	25	49	4	50	35	186	349
Missing or unknown	0	4	0	20	0	0	24
Fencing around garden							
Yes	97	102	22	404	45	239	909
No	38	30	4	85	64	117	338
No garden [b]	4	29	8	73	0	7	121
Missing or unknown	2	4	0	4	1	0	11
Garden borders on woods							
Yes	15	29	7	102	44	229	426
No	112	103	19	375	65	126	800

No garden [b]	12	29	8	88	0	8	145
Missing or unknown	2	4	0	1	1	0	8
Garden visited by wild animals							
Yes	35	36	7	104	44	250	476
No	98	91	17	353	60	99	718
No garden [b]	4	26	8	76	0	7	121
Missing or unknown	4	12	2	33	6	7	64
How much of garden is dense trees							
No woods [b]	43	56	12	165	50	200	526
Less than half of it	68	85	18	274	40	119	604
Half or more	25	20	4	104	17	43	213
Missing or unknown	5	4	0	23	3	1	36
Neighbourhood greater than 50% woods							
Yes	24	28	13	99	53	156	373
No	111	120	18	387	56	200	892
Missing or unknown [b]	6	17	3	80	1	7	114
Bird feeder within reach of wild animals							
Yes	29	57	2	128	29	182	427
No	108	88	25	363	81	97	762
No bird feeder [b]	3	15	7	70	0	84	179
Missing or unknown	1	5	0	5	0	0	11
Lifestyle & Outside Activities							
Pets spend time outside							
Yes	63	45	11	240	58	208	625
No [b]	78	115	23	326	52	155	749
Missing or unknown	0	5	0	0	0	0	5
Outside leisure activities							
Biking	55	87	13	230	64	164	613
Camping	23	8	8	38	11	92	180
Fishing	11	2	3	18	6	71	111
Foraging	2	24	5	219	22	254	526
Gardening	75	101	20	310	74	320	900
Hiking	74	76	16	324	82	265	837
Hunting	0	4	0	9	4	39	56
Sports	46	60	4	136	53	88	387
Picnicking	24	21	7	92	57	189	390

Outdoor play (children)	40	34	11	128	70	129	412
Other	20	15	5	21	11	66	138
None [b]	6	4	2	27	0	0	39
Missing	0	4	0	0	0	0	4
Time spent outside by season [c,d], mean (hours per week) (SD)							
Spring – leisure activity	18.6 (13.0)	11.7 (7.9)	17.5 (14.0)	20.0 (15.6)	18.2 (10.7)	20.1 (14.6)	18.7 (14.2)
Spring – occupational activity	6.3 (13.1)	1.1 (4.2)	3.7 (10.0)	5.4 (11.9)	5.0 (9.3)	4.7 (11.3)	4.7 (11.1)
Summer – leisure activity	26.8 (17.6)	16.5 (12.1)	26.3 (18.9)	29.1 (19.4)	26.3 (15.5)	30.9 (18.6)	27.6 (18.5)
Summer – occupational activity	9.0 (17.3)	1.6 (6.1)	4.5 (12.5)	6.7 (14.2)	6.5 (12.2)	5.6 (13.6)	6.0 (13.6)
Autumn – leisure activity	17.6 (12.0)	11.2 (9.1)	15.9 (12.8)	21.8 (16.3)	18.1 (11.4)	19.5 (14.5)	19.1 (14.6)
Autumn – occupational activity	5.8 (11.6)	1.0 (3.8)	3.1 (7.9)	5.4 (11.8)	5.6 (10.8)	4.5 (11.0)	4.6 (10.8)
Winter – leisure activity	11.3 (9.2)	7.2 (6.4)	9.0 (7.5)	12.2 (10.9)	12.3 (9.8)	11.1 (10.0)	11.1 (10.0)
Winter – occupational activity	3.8 (8.3)	0.7 (2.9)	2.2 (5.5)	4.2 (9.5)	3.7 (7.6)	3.3 (8.9)	3.4 (8.5)

[a] Outdoor occupations included construction, farming, forestry, land surveying, landscaping, park or wildlife management, railroad work, utility line work, and other outdoor work.

[b] Includes "Not applicable."

[c] Seasons were defined as: spring (April to June), summer (July to September), autumn (October to December), winter (January to March).

[d] Seasonal variables were combined in the regression analysis (occupational + summer leisure).

Disclaimer/Publisher's Note: The statements, opinions and data contained in all publications are solely those of the individual author(s) and contributor(s) and not of MDPI and/or the editor(s). MDPI and/or the editor(s) disclaim responsibility for any injury to people or property resulting from any ideas, methods, instructions or products referred to in the content.
